# Supplementary material for: A Personal Breast Cancer Risk Stratification Model Using Common Variants and Environmental Risk Factors in Japanese Females
Source: Cancers (Basel). 2021 Jul 28;13(15):3796. doi: 10.3390/cancers13153796 (PMC8345053; doi:10.3390/cancers13153796)
Supplement: Supplementary file 1 [file cancers-13-03796-s001.zip › cancers-1279159-Supplementary.pdf]

cancers

MDPI

Supplementary material

A Personal Breast Cancer Risk Stratification Model Using Common Variants and Environmental Risk Factors in Japanese Females

Isao Oze, Hidemi Ito, Yumiko Kasugai, Taiki Yamaji, Yuko Kijima, Tomotaka Ugai, Yoshio Kasuga, Tomoyo K. Ouellette, Yukari Taniyama, Yuriko N. Koyanagi, Issei Imoto, Shoichiro Tsugane, Chihaya Koriyama, Motoki Iwasaki and Keitaro Matsuo

Table S1. List of breast cancer-associated SNPs reported in GWAS studies.

SNP	Chr	Region	Position	Reference /Risk Allele	Gene
rs11249433	chr1	1p11.2	121538815	C/T	EMBP1
rs11552449	chr1	1p13.2	113905767	C/T	DCLRE1B
rs616488	chr1	1p36.22	10506158	C/T	PEX14
rs12405132	chr1	1q21.1	145790097	C/T	RNF115
rs12048493	chr1	1q21.2	149955122	A/C	OTUD7B
rs6678914	chr1	1q32.1	202218048	A/G	LGR6
rs4245739	chr1	1q32.1	204549714	A/C	MDM4
rs72755295	chr1	1q43	241870961	A/A	EXO1
rs12710696	chr2	2p24.1	19121042	C/T	LINC01376
rs4849887	chr2	2q14.2	120487546	C/T	LINC01101 - AC073257.2
rs1550623	chr2	2q31.1	173348166	C/T	CDCA7
rs2016394	chr2	2q31.1	172108243	C/T	DLX2-DT
rs1045485	chr2	2q33.1	201284866	G/G	CASP8
rs10931936	chr2	2q33.1	201279205	T/C	CASP8
rs16857609	chr2	2q35	217431785	C/T	DIRC3
rs13387042	chr2	2q35	217041109	A/G	AC007749.1 - RN7SKP43
rs1053338	chr3	3p14.1	63982224	A/G	ATXN7
rs6796502	chr3	3p21.31	46825376	A/G	PRSS50 - PRSS42
rs4973768	chr3	3p24.1	27374522	C/T	SLC4A7
rs12493607	chr3	3p24.1	30641447	C/G	TGFBR2

rs6762644	chr3	3p26.1	4700592	A/G	ITPR1
rs9790517	chr4	4q24	105163621	C/T	TET2
rs6828523	chr4	4q34.1	174925275	A/C	ADAM29
rs7697216	chr4	4q34.1	174906885	C/T	intergenic
rs10941679	chr5	5p12	44706396	A/G	AC093292.1 - RN7SL383P
rs7716600	chr5	5p12	44874903	A/C	MRPS30
rs9790879	chr5	5p12	44899783	C/T	intergenic
rs2012709	chr5	5p13.3	32567626	C/T	SUB1
rs13162653	chr5	5p15.1	16187419	G/T	AC092335.1 - NACAP6
rs2736108	chr5	5p15.33	1297373	A/G	TERT - MIR4457
rs10069690	chr5	5p15.33	1279675	C/T	TERT
rs7726159	chr5	5p15.33	1282204	A/C	TERT
rs889312	chr5	5q11.2	56736057	A/C	MAP3K1
rs1353747	chr5	5q11.2	59041654	T/T	PDE4D
rs10472076	chr5	5q11.2	58888234	C/T	RAB3C
rs7707921	chr5	5q14.2	82242227	A/T	ATG10
rs1432679	chr5	5q33.3	158817075	C/T	EBF1
rs9257408	chr6	6p22.1	28958443	G/G	LINC01556 - KRT18P1
rs204247	chr6	6p23	13722291	A/G	RANBP9
rs11242675	chr6	6p25.3	1318643	C/T	FOXQ1
rs17529111	chr6	6q14.1	81418669	C/T	AL590824.1 - TENT5A
rs2180341	chr6	6q22.33	127279485	A/G	RNF146
rs3757318	chr6	6q25.1	151592978	A/G	CCDC170
rs2046210	chr6	6q25.1	151627231	T/C	CCDC171
rs12662670	chr6	6q25.1	151597721	G/T	CCDC170
rs9485370	chr6	6q25.1	149285665	G/T	TAB2
rs6964587	chr7	7q21.2	92001306	G/T	AKAP9
rs4593472	chr7	7q32.3	130982362	C/T	LINC-PINT, AC016831.7
rs720475	chr7	7q35	144377836	A/G	ARHGEF5
rs13365225	chr8	8p11.23	37000965	A/G	AC090453.1 - AC092818.1
rs9693444	chr8	8p12	29652100	A/C	RPL17P33 - LINC00589
rs2943559	chr8	8q21.13	75505702	A/G	HNF4G
rs6472903	chr8	8q21.13	75318066	G/T	CASC9
rs13267382	chr8	8q23.3	116197325	A/G	LINC00536
rs13281615	chr8	8q24.21	127343372	G/A	CASC8, POU5F1B, PCAT1

rs11780156	chr8	8q24.21	128182395	C/T	MYC, MIR1208
rs1011970	chr9	9p21.3	22062135	G/T	CDKN2A, CDKN2B
rs10759243	chr9	9q31.2	107543834	A/C	PPIAP88 - RNU6-996P
rs865686	chr9	9q31.2	108126198	G/T	KLF4, ACTL7A, RAD23B
rs11814448	chr10	10p12.31	22026914	A/C	DNAJC1
rs7072776	chr10	10p12.31	21744013	A/G	DNAJC1, MLLT10
rs2380205	chr10	10p15.1	5844771	C/T	ANKRD16, FBXO18
rs10822013	chr10	10q21.2	62492218	C/T	ZNF365
rs10995190	chr10	10q21.2	62518923	A/G	ZNF365
rs704010	chr10	10q22.3	79081391	A/G	ZMIZ1
rs7904519	chr10	10q25.2	113014168	A/G	TCF7L2
rs11199914	chr10	10q26.12	121334387	C/T	LINC01153 - RN7SKP167
rs11200014	chr10	10q26.13	121575416	A/G	FGFR2
rs1219648	chr10	10q26.13	121586676	A/G	FGFR2
rs2420946	chr10	10q26.13	121591810	C/T	FGFR2
rs2981578	chr10	10q26.13	121580797	A/G	FGFR2
rs2981579	chr10	10q26.13	121577821	G/A	FGFR2
rs2981582	chr10	10q26.13	121592803	C/T	FGFR2
rs3817198	chr11	11p15.5	1887776	C/T	LSP1
rs3903072	chr11	11q13.1	65815595	A/C	DKFZp761E198, OVOL1, SNX32, CFL1, MUS81
rs614367	chr11	11q13.3	69513996	C/T	FGF3, MYEOV, CCND1, ORAOV1, FGF19, FGF4
rs554219	chr11	11q13.3	69516874	C/G	LINC01488 - CCND1
rs75915166	chr11	11q13.3	69564393	A/C	LINC01488 - CCND1
rs78540526	chr11	11q13.3	69516650	C/T	LINC01488 - CCND1
rs11820646	chr11	11q24.3	129591276	C/T	RPS27P20 - AP003500.2
rs7107217	chr11	11q24.3	129603795	A/C	TMEM45B, BARX2
rs10771399	chr12	12p11.22	28002147	A/G	PTHLH
rs12422552	chr12	12p13.1	14260997	C/G	GNAI2P1 - RPL30P11
rs17356907	chr12	12q22	95633983	A/G	NTN4
rs1292011	chr12	12q24.21	115398717	C/T	AC078880.2 - AC009803.2
rs11571833	chr13	13q13.1	32398489	A/A	BRCA2
rs2236007	chr14	14q13.3	36663564	A/G	PAX9
rs8009944	chr14	14q24.1	68572871	A/C	RAD51B

rs2588809	chr14	14q24.1	68193711	C/T	RAD51L1
rs999737	chr14	14q24.1	68567965	C/T	RAD51L1
rs941764	chr14	14q32.11	91374725	A/G	CCDC88C
rs11627032	chr14	14q32.12	92637727	C/T	RIN3
rs3803662	chr16	16q12.1	52552429	C/T	TNRC9, LOC643714
rs12443621	chr16	16q12.1	52514125	A/G	TOX3
rs17271951	chr16	16q12.1	52504128	C/T	TOX3
rs8051542	chr16	16q12.1	52500255	C/T	TOX3
rs4784227	chr16	16q12.1	52565276	T/C	TOX3
rs11075995	chr16	16q12.2	53821379	A/T	FTO
rs17817449	chr16	16q12.2	53779455	G/T	FTO
rs13329835	chr16	16q23.2	80616908	A/G	CDYL2
rs6504950	chr17	17q22	54979110	A/G	COX11
rs1156287	chr17	17q22	54999438	C/T	STXBP4
rs745570	chr17	17q25.3	79807926	C/T	AC100791.2 - LINC01977
rs1436904	chr18	18q11.2	26990703	G/T	CHST9
rs527616	chr18	18q11.2	26757460	C/G	AQP4-AS1
rs6507583	chr18	18q12.3	44819625	A/G	SETBP1
rs8170	chr19	19p13.11	17278895	C/C	ANKLE1, C19orf62, ABHD8
rs2363956	chr19	19p13.11	17283315	G/T	ANKLE1
rs4808801	chr19	19p13.11	18460331	A/G	ELL
rs3760982	chr19	19q13.31	43782361	A/G	KCNN4 - LYPD5
rs2823093	chr21	21q21.1	15148511	A/G	NRIP1
rs17879961	chr22	22q12.1	28725099	T/T	CHEK2
rs132390	chr22	22q12.2	29225488	T/T	EMID1
rs6001930	chr22	22q13.1	40480230	C/T	MKL1

Table S2. ORs of genetic risk groups stratified by menopausal status.

			Crude model*				Adjusted model**			
Risk group	Cases	Controls	OR	95% CI	p-value	trend p	OR	95% CI	p-value	trend p
Premenopause										
Low***	78	254	Reference			2.47×10^{-11}	Reference			1.47×10^{-11}
Moderate***	429	704	1.97	(1.49- 2.62)	2.77×10^{-6}		2.03	(1.52- 2.72)	1.86×10^{-6}	
High***	84	65	4.05	(2.67- 6.15)	5.13×10^{-11}		4.27	(2.78- 6.56)	3.22×10^{-11}	
Postmenopause										
Low***	110	245	Reference			3.36×10^{-7}	Reference			7.66×10^{-7}
Moderate***	502	754	1.46	(1.13- 1.88)	3.60×10^{-3}		1.46	(1.13- 1.90)	4.14×10^{-3}	
High***	99	80	2.72	(1.87- 3.96)	1.66×10^{-7}		2.69	(1.83- 3.95)	4.23×10^{-7}	

OR; odds ratio, CI; confidence interval

* Age was included in the crude model.

** Age, BMI, ethanol intake, smoking, physical activity, family history of breast cancer, age at menarche, parity, number of births, age at first birth, breastfeeding and hormone therapy were included in the adjusted model.

***Genetic risk group was defined by number of risk alleles. Number of risk alleles of 0-10, 11-16 and 17-28 were defined as low, moderate and high risk group, respectively.

Table 3. C-statistics of genetic, environmental and inclusive risk models stratified by menopausal status.

Menopausal status	Genetic			Environment			Inclusive			
	C-statistics	95%	CI	C-statistics	95%	CI	C-statistics	95%	CI	p-value
Premenopause	0.652	(0.625	-0.680)	0.611	(0.582	-0.640)	0.677	(0.650	-0.704)	5.53×10^{-8}
Postmenopause	0.621	(0.594	-0.647)	0.625	(0.598	-0.651)	0.663	(0.638	-0.689)	1.44×10^{-5}

Genetic risk group was included in the Genetic model.

Age, BMI, ethanol intake, smoking, physical activity, family history of breast cancer, age at menarche, parity, number of births, age at first birth, breastfeeding and hormone therapy were included in the Environment model.

All variables in Genetic model and Environment model were included in the Inclusive model.

p-values were calculated by testing differences between the Environment model and Inclusive model.

Table 4. C-statistics of genetic risk score models including 14, 22 and 42 SNPs.

No. of SNPs included in risk score models	Pooled			Nagano			Kagoshima			Aichi		
	C-statistics	95%	CI	C-statistics	95%	CI	C-statistics	95%	CI	C-statistics	95%	CI
14 SNPs	0.633	(0.614-	0.652)	0.605	(0.566-	0.645)	0.609	(0.560-	0.657)	0.604	(0.579-	0.630)
22 SNPs	0.636	(0.617-	0.655)	0.602	(0.562-	0.642)	0.644	(0.597-	0.690)	0.601	(0.576-	0.627)
42 SNPs	0.636	(0.617-	0.655)	0.610	(0.570-	0.650)	0.648	(0.601-	0.694)	0.593	(0.567-	0.618)
p-value*	0.772			0.861			0.159			0.688		

*P-values were calculated comparing c-statistics of three genetic risk models.

Table S5. C-statistics of three levels of genetic risk score model, risk score model and allelic risk model.

**Risk model**	**Pooled**			**Nagano**			**Kagoshima**			**Aichi**		
	C-statistics	0.95	CI	C-statistics	0.95	CI	C-statistics	0.95	CI	C-statistics	0.95	CI
Risk score model in 3 levels	0.614	(0.595-	0.632)	0.560	(0.527-	0.593)	0.570	(0.529-	0.610)	0.572	(0.551-	0.593)
Risk score model	0.633	(0.614-	0.652)	0.605	(0.566-	0.645)	0.609	(0.560-	0.657)	0.604	(0.579-	0.630)
Allelic risk model	0.639	(0.620-	0.658)	0.636	(0.598-	0.675)	0.636	(0.590-	0.683)	0.614	(0.589-	0.640)
Risk score model vs allelic risk model	p=0.202			p=0.150			p=0.268			p=0.187		

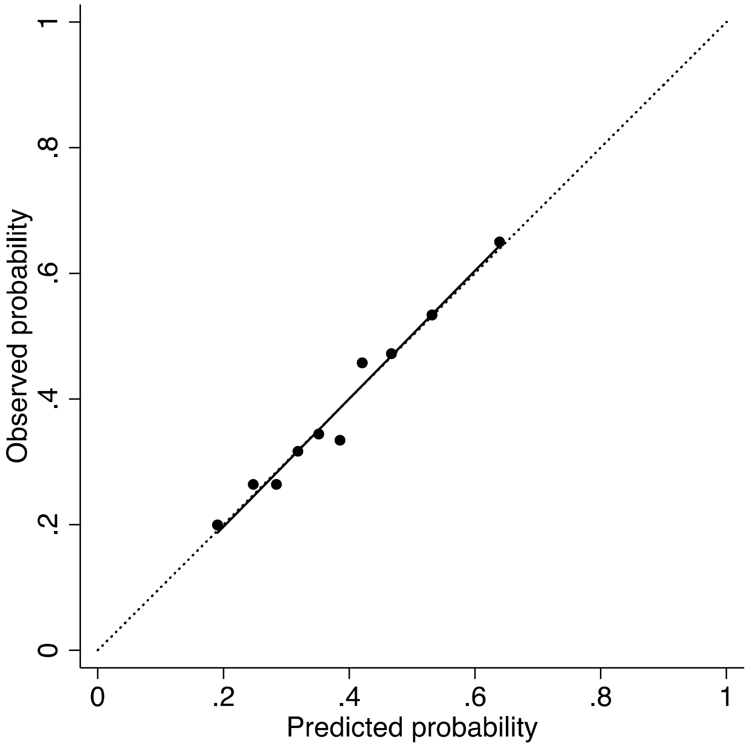

Figure S1. Caliblation plot of inclusive model in the total population. Predicted and observed probabilities within deciles of predicted probability were plotted. Dotted line represents the ideal caliblation line and the solid line shows the linear caliblation line for the risk model.
